# Supplementary material for: Identification and molecular characterization of mutations in nucleocapsid phosphoprotein of SARS-CoV-2
Source: PeerJ. 2021 Jan 4;9:e10666. doi: 10.7717/peerj.10666 (PMC7789862; doi:10.7717/peerj.10666)
Supplement: Supplemental Information 1 — The table shows the protein accession identifier numbers of N prtoein used in this study. All of these sequences are downloaded from NCBI virus database. The list contains a total of 4163 accession numbers. All of these sequences were reported from USA. [file peerj-09-10666-s001.docx]

Supplementary table 1: List of protein accession number used in this study (total 4163)

| YP_009724397 | QKS90955 | QKV07251 | QKG89446 | QJU70553 | QJE38722 | QIZ64740 |
| --- | --- | --- | --- | --- | --- | --- |
| QKV35192 | QKS90967 | QKV07263 | QKG89458 | QJU70577 | QJE38734 | QIZ12971 |
| QKV35204 | QKS90979 | QKV07275 | QKG89470 | QJU70589 | QJE38746 | QIZ12983 |
| QKV35228 | QKS90991 | QKV07287 | QKG89482 | QJV21790 | QJE38758 | QIZ13019 |
| QKV35240 | QKS91003 | QKV07299 | QKG89494 | QJV21802 | QJE38770 | QIZ13031 |
| QKV35252 | QKS91015 | QKV07311 | QKG89506 | QJV21814 | QJE38782 | QIZ13043 |
| QKV35275 | QKS91027 | QKV07323 | QKG89518 | QJV21826 | QJE38794 | QIZ13055 |
| QKV35287 | QKS91039 | QKV07335 | QKG89530 | QJV21838 | QJE38806 | QIZ13115 |
| QKV35299 | QKS91063 | QKV07347 | QKG89542 | QJV21850 | QJE38818 | QIZ13127 |
| QKV35311 | QKS91075 | QKV07359 | QKG89554 | QJV21862 | QJE38830 | QIZ13163 |
| QKV35323 | QKS91087 | QKV07371 | QKG89566 | QJV21874 | QJE38842 | QIZ13175 |
| QKV35335 | QKS91099 | QKV07383 | QKG89578 | QJV21886 | QJE38854 | QIZ13187 |
| QKV35347 | QKS91111 | QKV07407 | QKG89590 | QJV21898 | QJE38866 | QIZ13199 |
| QKV35359 | QKT21082 | QKV07419 | QKG89602 | QJV58836 | QJE38878 | QIZ13247 |
| QKV35371 | QKT21094 | QKV07431 | QKG89614 | QJW28204 | QJE38890 | QIZ13271 |
| QKV35383 | QKT21106 | QKV07443 | QKG89626 | QJW28216 | QJE38902 | QIZ13283 |
| QKV35395 | QKT21118 | QKV07455 | QKG89638 | QJW28240 | QJE38914 | QIZ13307 |
| QKV35407 | QKT21130 | QKV07467 | QKG89650 | QJW28252 | QJE38926 | QIZ13319 |
| QKV35419 | QKT21142 | QKV07479 | QKG89662 | QJW28264 | QJE38938 | QIZ13331 |
| QKV35431 | QKT21154 | QKV07503 | QKG89674 | QJW28276 | QJE38950 | QIZ13343 |
| QKV35443 | QKT21166 | QKV07515 | QKG89686 | QJW28288 | QJE38962 | QIZ13355 |
| QKV35455 | QKT21178 | QKV07527 | QKG89710 | QJW28300 | QJE38974 | QIZ13367 |
| QKV35467 | QKT21190 | QKV07539 | QKG89722 | QJW28312 | QJE38986 | QIZ13391 |
| QKV35479 | QKT21202 | QKV07551 | QKG89734 | QJW28336 | QJE38998 | QIZ13427 |
| QKV35491 | QKT21214 | QKV07563 | QKG89746 | QJW28348 | QJE39034 | QIZ13451 |
| QKV35503 | QKT21226 | QKV07575 | QKG89770 | QJW28360 | QJE39070 | QIZ13475 |
| QKV35515 | QKT21238 | QKV07587 | QKG89782 | QJW28372 | QJD07696 | QIZ13487 |
| QKV35527 | QKT21250 | QKV07599 | QKG89794 | QJW28384 | QJD47054 | QIZ13499 |
| QKV35539 | QKT21262 | QKV07611 | QKG89806 | QJW28396 | QJD47066 | QIZ13511 |
| QKV35551 | QKT21274 | QKV07623 | QKG89818 | QJW28408 | QJD47078 | QIZ13523 |
| QKV35563 | QKT21286 | QKV07635 | QKG89830 | QJW28420 | QJD47090 | QIZ13535 |
| QKV35575 | QKT21298 | QKV07647 | QKG89842 | QJW28432 | QJD47102 | QIZ13547 |
| QKV35587 | QKT21310 | QKV07659 | QKG89866 | QJW28444 | QJD47114 | QIZ13583 |
| QKV35599 | QKT21322 | QKV07671 | QKG89878 | QJW28456 | QJD47126 | QIZ13595 |
| QKV35611 | QKT21334 | QKV07695 | QKG89890 | QJW28468 | QJD47138 | QIZ13607 |
| QKV35623 | QKT21346 | QKV07707 | QKG89914 | QJW28480 | QJD47150 | QIZ13631 |
| QKV35635 | QKT21358 | QKV07719 | QKG89926 | QJW28492 | QJD47162 | QIZ13665 |
| QKV35647 | QKT21370 | QKV07731 | QKG89938 | QJW28504 | QJD47174 | QIZ13677 |
| QKV35659 | QKT21382 | QKV07743 | QKG89950 | QJW28516 | QJD47198 | QIZ13689 |
| QKV35671 | QKT21394 | QKV07755 | QKG89962 | QJW28528 | QJD47210 | QIZ13701 |
| QKV35683 | QKT21406 | QKV07767 | QKG89974 | QJW28540 | QJD47222 | QIZ13713 |
| QKV35695 | QKT21418 | QKV07779 | QKG90010 | QJW28552 | QJD47234 | QIZ13725 |
| QKV35707 | QKT21430 | QKV07791 | QKG90022 | QJW28564 | QJD47246 | QIZ13737 |
| QKV35719 | QKT21442 | QKV07803 | QKG90034 | QJW28576 | QJD47258 | QIZ13749 |
| QKV35731 | QKU28446 | QKV07815 | QKG90046 | QJW28588 | QJD47270 | QIZ13761 |
| QKV35755 | QKU28470 | QKV07827 | QKG90058 | QJW28600 | QJD47282 | QIZ13773 |
| QKV35767 | QKU28506 | QKV07839 | QKG90082 | QJW28612 | QJD47294 | QIZ13785 |
| QKV35779 | QKU28518 | QKV07851 | QKG90094 | QJW28624 | QJD47306 | QIZ13797 |
| QKV35791 | QKU28530 | QKV07863 | QKG90106 | QJW28636 | QJD47318 | QIZ13809 |
| QKV35803 | QKU28542 | QKV07875 | QKG90118 | QJW28648 | QJD47330 | QIZ13821 |
| QKV35815 | QKU28554 | QKV07887 | QKG90130 | QJW28660 | QJD47342 | QIZ13833 |
| QKV35827 | QKU28578 | QKV07899 | QKG90154 | QJW28672 | QJD47354 | QIZ13845 |
| QKV35839 | QKU28590 | QKV07911 | QKG90166 | QJU11429 | QJD47366 | QIZ13857 |
| QKV35851 | QKU28602 | QKV07923 | QKG90178 | QJU11441 | QJD47378 | QIZ13881 |
| QKV35863 | QKU28614 | QKV07935 | QKG90190 | QJU11453 | QJD47390 | QIZ13893 |
| QKV35875 | QKU28626 | QKV07947 | QKG90202 | QJU11465 | QJD47402 | QIZ13929 |
| QKV35887 | QKU28638 | QKV07959 | QKG90226 | QJU11477 | QJD47414 | QIZ13941 |
| QKV35899 | QKU28650 | QKV07971 | QKG90250 | QJU11489 | QJD47426 | QIZ13953 |
| QKV35911 | QKU28662 | QKV07983 | QKG90262 | QJU11501 | QJD47438 | QIZ13965 |
| QKV35923 | QKU28674 | QKV07995 | QKG90274 | QJU11513 | QJD47450 | QIZ14025 |
| QKV35935 | QKU28686 | QKV08007 | QKG90286 | QJU11525 | QJD47462 | QIZ14037 |
| QKV35947 | QKU28698 | QKV08019 | QKG90298 | QJU11537 | QJD47474 | QIZ14049 |
| QKV35959 | QKU28710 | QKV08031 | QKG90322 | QJU11549 | QJD47498 | QIZ14085 |
| QKV35971 | QKU28722 | QKV08043 | QKG90334 | QJU11561 | QJD47510 | QIZ14097 |
| QKV35983 | QKU28734 | QKV08055 | QKG90346 | QJU11585 | QJD47522 | QIZ14109 |
| QKV35995 | QKU28746 | QKV08067 | QKG90370 | QJU11597 | QJD47534 | QIZ14121 |
| QKV36007 | QKU28758 | QKV08079 | QKG90382 | QJU11609 | QJD47546 | QIZ14133 |
| QKV36019 | QKU28770 | QKV08091 | QKG90394 | QJU11621 | QJD47558 | QIZ14145 |
| QKV36031 | QKU28782 | QKV08103 | QKG90406 | QJU11633 | QJD47570 | QIZ14169 |
| QKV36043 | QKU28794 | QKV08115 | QKG90418 | QJU11645 | QJD47582 | QIZ14181 |
| QKV36055 | QKU28806 | QKV08127 | QKG90430 | QJU11657 | QJD47606 | QIZ14193 |
| QKV36067 | QKU28818 | QKV08139 | QKG90442 | QJU11808 | QJD47630 | QIZ14217 |
| QKV36079 | QKU28830 | QKV08151 | QKG90454 | QJT43412 | QJD47642 | QIZ14229 |
| QKV36091 | QKU28842 | QKV08163 | QKG90466 | QJS54534 | QJD47654 | QIZ14241 |
| QKV36103 | QKU28854 | QKV08175 | QKG90478 | QJS54546 | QJD47678 | QIZ14253 |
| QKV36115 | QKU28866 | QKV08187 | QKG90502 | QJS54558 | QJD47690 | QIZ14277 |
| QKV36127 | QKU28878 | QKV08199 | QKG90514 | QJS54570 | QJD47702 | QIZ14289 |
| QKV36139 | QKU28890 | QKV08211 | QKG90526 | QJS54582 | QJD47714 | QIZ14301 |
| QKV36151 | QKU28902 | QKV08223 | QKG90538 | QJS54594 | QJD47915 | QIZ14313 |
| QKV36163 | QKU28914 | QKV08235 | QKG90550 | QJS54606 | QJD47927 | QIZ14325 |
| QKV36175 | QKU28938 | QKV08247 | QKG90562 | QJS54618 | QJD47939 | QIZ14337 |
| QKV36187 | QKU28950 | QKV08259 | QKG90574 | QJS54630 | QJD47951 | QIZ14349 |
| QKV36199 | QKU28962 | QKV08271 | QKG90598 | QJS54642 | QJD47963 | QIZ14373 |
| QKV36211 | QKU28974 | QKV08283 | QKG90610 | QJS54654 | QJD47975 | QIZ14469 |
| QKV36223 | QKU28986 | QKV08295 | QKG90622 | QJS54666 | QJD47987 | QIZ14481 |
| QKV36235 | QKU28998 | QKV08307 | QKG90634 | QJS54690 | QJD47999 | QIZ14493 |
| QKV36247 | QKU29010 | QKV08319 | QKG90646 | QJS54702 | QJD48011 | QIZ14505 |
| QKV36259 | QKU29022 | QKV08331 | QKG90658 | QJS54714 | QJD48023 | QIZ14517 |
| QKV36271 | QKU29034 | QKV08343 | QKG90670 | QJS54750 | QJD48035 | QIZ14529 |
| QKV36283 | QKU29046 | QKV08355 | QKG90682 | QJS54762 | QJD48047 | QIZ14541 |
| QKV36295 | QKU29058 | QKV08367 | QKG90694 | QJS54786 | QJD48059 | QIZ14589 |
| QKV36307 | QKU29070 | QKV08379 | QKG90706 | QJS54798 | QJD48071 | QIZ14601 |
| QKV36319 | QKU29082 | QKV08391 | QKG90718 | QJS54810 | QJD48083 | QIZ14613 |
| QKV36331 | QKU29094 | QKV08403 | QKG90730 | QJS54822 | QJD48095 | QIZ14625 |
| QKV36343 | QKU30337 | QKV08415 | QKG90742 | QJS54846 | QJD48119 | QIZ14637 |
| QKV36355 | QKU30349 | QKV08427 | QKG90778 | QJS54858 | QJD48131 | QIZ14661 |
| QKV36367 | QKU30361 | QKV08439 | QKG90790 | QJS54882 | QJD48143 | QIZ14685 |
| QKV36379 | QKU30373 | QKV08451 | QKG90802 | QJS54894 | QJD48155 | QIZ14697 |
| QKV36391 | QKU30385 | QKV08463 | QKG90814 | QJS54906 | QJD48167 | QIZ14721 |
| QKV36403 | QKU30397 | QKV08475 | QKG90826 | QJS54918 | QJD48179 | QIZ14769 |
| QKV36415 | QKU30409 | QKV08487 | QKG90838 | QJS54930 | QJD48191 | QIZ14829 |
| QKV36427 | QKU30421 | QKV08499 | QKG90850 | QJS54942 | QJD48203 | QIZ14853 |
| QKV36439 | QKU30433 | QKV08511 | QKG90862 | QJS56543 | QJD48215 | QIZ14865 |
| QKV36451 | QKU30445 | QKV08523 | QKG90874 | QJS56555 | QJD48227 | QIZ14901 |
| QKV36463 | QKU30457 | QKV08535 | QKG90898 | QJS56567 | QJD48239 | QIZ14913 |
| QKV36475 | QKU30469 | QKV08547 | QKG90910 | QJS56579 | QJD48251 | QIZ14937 |
| QKV36487 | QKU30481 | QKV08559 | QKG90934 | QJS56591 | QJD48263 | QIZ14973 |
| QKV36499 | QKU30493 | QKV08571 | QKG90946 | QJS56603 | QJD48275 | QIZ14985 |
| QKV36511 | QKU30505 | QKV08583 | QKG90958 | QJS56615 | QJD48287 | QIZ15005 |
| QKV36523 | QKU30517 | QKV08595 | QKG90982 | QJS56627 | QJD48299 | QIZ15557 |
| QKV36547 | QKU30529 | QKV08607 | QKG90994 | QJS56639 | QJD48311 | QIZ15569 |
| QKV36559 | QKU30541 | QKV08619 | QKG91006 | QJS56651 | QJD48323 | QIZ15581 |
| QKV36571 | QKU30553 | QKV08631 | QKG91018 | QJS56663 | QJD48347 | QIZ15593 |
| QKV36583 | QKU30565 | QKV08643 | QKG91030 | QJS56675 | QJD48359 | QIZ15605 |
| QKV36595 | QKU30577 | QKV08655 | QKG91042 | QJS56687 | QJD48371 | QIZ15617 |
| QKV36607 | QKU30589 | QKV08667 | QKG91054 | QJS56699 | QJD48383 | QIZ15629 |
| QKV36619 | QKU30601 | QKV08679 | QKG91066 | QJS56711 | QJD48395 | QIZ15641 |
| QKV36631 | QKU30613 | QKV08691 | QKG91078 | QJS56723 | QJD48407 | QIZ15653 |
| QKV36643 | QKU30625 | QKV08703 | QKG91090 | QJS56735 | QJD48419 | QIZ15665 |
| QKV36655 | QKU30637 | QKV08715 | QKG91102 | QJS56747 | QJD48431 | QIZ15677 |
| QKV36667 | QKU30649 | QKV08727 | QKG91114 | QJS56759 | QJD48443 | QIZ15689 |
| QKV36679 | QKU30661 | QKV08739 | QKG91126 | QJS56771 | QJD48455 | QIZ15701 |
| QKV36691 | QKU30673 | QKV08751 | QKG91150 | QJS56783 | QJD48467 | QIZ15713 |
| QKV36703 | QKU30685 | QKV08763 | QKG91162 | QJS56795 | QJD48479 | QIZ15725 |
| QKV36715 | QKU30697 | QKV08775 | QKI30384 | QJS56807 | QJD48491 | QIZ15737 |
| QKV36727 | QKU30709 | QKV08787 | QKI30396 | QJS56819 | QJD48503 | QIZ15749 |
| QKV36739 | QKU30721 | QKV08799 | QKI30408 | QJS56831 | QJD48527 | QIZ15761 |
| QKV36751 | QKU30733 | QKV08811 | QKI30420 | QJS56843 | QJD48539 | QIZ15773 |
| QKV36775 | QKU30745 | QKV08823 | QKI30432 | QJS56855 | QJD48551 | QIZ15785 |
| QKV36787 | QKU30757 | QKV08835 | QKG64059 | QJS56867 | QJD48563 | QIZ15797 |
| QKV36799 | QKU30769 | QKV08859 | QKG64071 | QJS56879 | QJD48575 | QIZ15809 |
| QKV36811 | QKU30781 | QKV08871 | QKG64083 | QJS56891 | QJD48587 | QIZ15821 |
| QKV36823 | QKU30793 | QKV08883 | QKG81327 | QJS56903 | QJD48599 | QIZ15833 |
| QKV36835 | QKU30805 | QKV08895 | QKG81339 | QJS56915 | QJD48611 | QIZ15845 |
| QKV36847 | QKU30817 | QKV08907 | QKG81351 | QJS56927 | QJD48623 | QIZ15857 |
| QKV36859 | QKU30829 | QKV08919 | QKG81363 | QJS56939 | QJD48635 | QIZ15869 |
| QKV36871 | QKU30841 | QKV08931 | QKG81375 | QJS56951 | QJD48647 | QIZ15881 |
| QKV36883 | QKU30853 | QKV08943 | QKG81387 | QJS56963 | QJD48659 | QIZ15893 |
| QKV36895 | QKU30865 | QKV08955 | QKG81399 | QJS56975 | QJD48671 | QIZ15905 |
| QKV36907 | QKU30877 | QKV08967 | QKG81411 | QJS56987 | QJD48683 | QIZ15917 |
| QKV36919 | QKU30889 | QKV08979 | QKG81423 | QJS56999 | QJD48695 | QIZ15929 |
| QKV36931 | QKU30901 | QKV08991 | QKG81435 | QJS57011 | QJD48707 | QIZ15941 |
| QKV36943 | QKU30925 | QKV09003 | QKG81447 | QJS57023 | QJD48719 | QIZ15953 |
| QKV36955 | QKU30937 | QKV09015 | QKG81459 | QJS57035 | QJD48731 | QIZ15965 |
| QKV36967 | QKU30949 | QKV09027 | QKG81471 | QJS57047 | QJD48743 | QIZ15977 |
| QKV36979 | QKU30961 | QKV09039 | QKG81483 | QJS57059 | QJD48779 | QIZ15989 |
| QKV36991 | QKU30973 | QKV09051 | QKG81495 | QJS57071 | QJD48791 | QIZ16001 |
| QKV37003 | QKU30985 | QKV09063 | QKG81507 | QJS57083 | QJD48803 | QIZ16013 |
| QKV37039 | QKU30997 | QKV09075 | QKG81519 | QJS57095 | QJD48815 | QIZ16025 |
| QKV37051 | QKU31009 | QKV09087 | QKG81531 | QJS57107 | QJD48839 | QIZ16037 |
| QKV37063 | QKU31021 | QKV09099 | QKG81543 | QJS57119 | QJD48851 | QIZ16049 |
| QKV37075 | QKU31033 | QKV09111 | QKG81555 | QJS57131 | QJD48863 | QIZ16061 |
| QKV37087 | QKU31057 | QKV09123 | QKG81567 | QJS57143 | QJD48875 | QIZ16073 |
| QKV37099 | QKU31069 | QKV09135 | QKG81579 | QJS57155 | QJD48887 | QIZ16085 |
| QKV38757 | QKU31093 | QKV09147 | QKG81591 | QJS57167 | QJD48899 | QIZ16097 |
| QKV38769 | QKU31105 | QKV09159 | QKG81603 | QJS57179 | QJD48911 | QIZ16109 |
| QKV38805 | QKU31117 | QKV09171 | QKG81615 | QJS57191 | QJD48923 | QIZ16121 |
| QKV38817 | QKU31129 | QKV09195 | QKG81627 | QJS57203 | QJD48935 | QIZ16133 |
| QKV38829 | QKU31141 | QKV09231 | QKG81639 | QJS57215 | QJD48947 | QIZ16145 |
| QKV38877 | QKU31153 | QKV09243 | QKG81651 | QJS57227 | QJD48959 | QIZ16157 |
| QKV38889 | QKU31165 | QKV09255 | QKG81663 | QJS57251 | QJD48971 | QIZ16169 |
| QKV38901 | QKU31189 | QKS65472 | QKG81675 | QJS57263 | QJD48983 | QIZ16181 |
| QKV38913 | QKU31213 | QKS65496 | QKG81687 | QJS57275 | QJD48995 | QIZ16193 |
| QKV38925 | QKU31225 | QKS65508 | QKG81699 | QJS57299 | QJD49007 | QIZ16205 |
| QKV38937 | QKU31249 | QKS65532 | QKG81711 | QJS57311 | QJD49019 | QIZ16217 |
| QKV38960 | QKU31261 | QKS65544 | QKG81723 | QJS57323 | QJD49031 | QIZ16229 |
| QKV38972 | QKU31273 | QKS65556 | QKG81735 | QJR83229 | QJD49043 | QIZ16241 |
| QKV38984 | QKU31297 | QKS65568 | QKG81747 | QJR83241 | QJD49055 | QIZ16253 |
| QKV38996 | QKU31309 | QKS65580 | QKG81759 | QJR83253 | QJD49067 | QIZ16265 |
| QKV39008 | QKU31369 | QKS65592 | QKG81771 | QJR83265 | QJD49079 | QIZ16277 |
| QKV39032 | QKU31405 | QKS65604 | QKG81783 | QJR83277 | QJD49091 | QIZ16289 |
| QKV39044 | QKU31441 | QKS65616 | QKG81795 | QJR83289 | QJD49103 | QIZ16301 |
| QKV39056 | QKU31465 | QKS65628 | QKG81807 | QJR83301 | QJD49115 | QIZ16313 |
| QKV39068 | QKU31477 | QKS65640 | QKG81819 | QJR83313 | QJD49127 | QIZ16325 |
| QKV39080 | QKU31501 | QKS65652 | QKG81831 | QJR83325 | QJD49139 | QIZ16337 |
| QKV39092 | QKU31525 | QKS65664 | QKG81843 | QJR83337 | QJD49151 | QIZ16349 |
| QKV39104 | QKU31537 | QKS65676 | QKG81855 | QJR83349 | QJD49163 | QIZ16361 |
| QKV39116 | QKU31549 | QKS65688 | QKG81867 | QJR83361 | QJD49175 | QIZ16373 |
| QKV39128 | QKU31561 | QKS65700 | QKG81879 | QJR83373 | QJD49199 | QIZ16385 |
| QKV39140 | QKU31573 | QKS65712 | QKG81891 | QJR83385 | QJD49211 | QIZ16397 |
| QKV39152 | QKU31585 | QKS65724 | QKG81903 | QJR83397 | QJD49223 | QIZ16409 |
| QKV39164 | QKU31597 | QKS65736 | QKG81915 | QJR83421 | QJD49247 | QIZ16421 |
| QKV39176 | QKU31609 | QKS65748 | QKG81927 | QJR83433 | QJD49259 | QIZ16433 |
| QKV39188 | QKU31621 | QKS65760 | QKG81939 | QJR83445 | QJD23281 | QIZ16445 |
| QKV39200 | QKU31633 | QKS65772 | QKG81951 | QJR83457 | QJD23305 | QIZ16457 |
| QKV39212 | QKU31645 | QKS65784 | QKG81963 | QJR83469 | QJD23317 | QIZ16469 |
| QKV39224 | QKU31657 | QKS65796 | QKG81975 | QJR83481 | QJD23329 | QIZ16481 |
| QKV39248 | QKU31669 | QKS65808 | QKG81987 | QJR83493 | QJD23341 | QIZ16493 |
| QKV39271 | QKU31681 | QKS65820 | QKG81999 | QJR83505 | QJD23353 | QIZ16505 |
| QKV39283 | QKU31693 | QKS65832 | QKG82011 | QJR83517 | QJD23365 | QIX13683 |
| QKV39295 | QKU31705 | QKS65844 | QKG82023 | QJR83529 | QJD23377 | QIX13695 |
| QKV39307 | QKU31717 | QKS65856 | QKG82035 | QJR83541 | QJD23401 | QIX13707 |
| QKV39319 | QKU31729 | QKS65868 | QKG82047 | QJR83553 | QJD23413 | QIX13719 |
| QKV39331 | QKU31741 | QKS65880 | QKG86513 | QJR83565 | QJD23425 | QIX13731 |
| QKV39343 | QKU31753 | QKS65892 | QKG86525 | QJR83577 | QJD23437 | QIX13755 |
| QKV39355 | QKU31765 | QKS65904 | QKG86537 | QJR83589 | QJD23449 | QIX13767 |
| QKV39367 | QKU31789 | QKS65916 | QKG86549 | QJR83601 | QJD23461 | QIX13779 |
| QKV39379 | QKU31801 | QKS65928 | QKG27861 | QJR83613 | QJD23485 | QIX13791 |
| QKV39391 | QKU31813 | QKS65940 | QKG27873 | QJR83625 | QJD23497 | QIX13803 |
| QKV39403 | QKU31825 | QKS65952 | QKG27885 | QJR83637 | QJD23509 | QIX13815 |
| QKV39415 | QKU31837 | QKS65964 | QKG27897 | QJR83661 | QJD23521 | QIX13827 |
| QKV39427 | QKU31849 | QKS65976 | QKG27909 | QJR83673 | QJD23532 | QIX13839 |
| QKV39439 | QKU31873 | QKS65988 | QKG27921 | QJR83685 | QJD23545 | QIX13851 |
| QKV39451 | QKU31885 | QKS66000 | QKG28068 | QJR83697 | QJD23557 | QIX13863 |
| QKV39463 | QKU31897 | QKS66012 | QKF30870 | QJR83709 | QJD23581 | QIX13875 |
| QKV39475 | QKU31909 | QKS66024 | QKE61644 | QJR83721 | QJD23593 | QIX13887 |
| QKV39487 | QKU31921 | QKS66036 | QKE61656 | QJR83733 | QJD23605 | QIX13899 |
| QKV39499 | QKU31933 | QKS66048 | QKE49108 | QJR83757 | QJD23617 | QIX13911 |
| QKV39511 | QKU31945 | QKS66060 | QKE49120 | QJR83769 | QJD23629 | QIX13923 |
| QKV39523 | QKU31969 | QKS66072 | QKE49132 | QJR84557 | QJD23641 | QIX13935 |
| QKV39535 | QKU32005 | QKS66084 | QKE49144 | QJR84569 | QJD23653 | QIX13947 |
| QKV39547 | QKU32017 | QKS66096 | QKE49156 | QJR84593 | QJD23665 | QIX13959 |
| QKV39571 | QKU32029 | QKS66108 | QKE49168 | QJR84617 | QJD23677 | QIX13971 |
| QKV39583 | QKU32041 | QKS66120 | QKE49192 | QJR84653 | QJD23689 | QIX13983 |
| QKV39595 | QKU32053 | QKS66132 | QKE49204 | QJR84689 | QJD23701 | QIX13995 |
| QKV39607 | QKU32077 | QKS66144 | QKE49216 | QJR84737 | QJD23725 | QIX14007 |
| QKV39619 | QKU32089 | QKS66156 | QKE49228 | QJR84797 | QJD23737 | QIX14019 |
| QKV39631 | QKU32101 | QKS66168 | QKE49240 | QJR84893 | QJD23749 | QIX14031 |
| QKV39643 | QKU32113 | QKS66180 | QKE49252 | QJR84905 | QJD23761 | QIX14043 |
| QKV39655 | QKU32125 | QKS66192 | QKE49264 | QJR84965 | QJD23773 | QIV64973 |
| QKV39667 | QKU32137 | QKS66204 | QKE49276 | QJR84989 | QJD23797 | QIV64985 |
| QKV39679 | QKU32149 | QKS66216 | QKE49300 | QJR85001 | QJD23809 | QIV64997 |
| QKV39691 | QKU32161 | QKS66228 | QKE49324 | QJR96406 | QJD23821 | QIV14980 |
| QKV39703 | QKU32173 | QKS66252 | QKE49336 | QJR96418 | QJD23833 | QIV15028 |
| QKV39715 | QKU32185 | QKS66264 | QKE49348 | QJR96430 | QJD23845 | QIV15040 |
| QKV39727 | QKU32209 | QKS66276 | QKE49360 | QJQ82632 | QJD23869 | QIV15052 |
| QKV39739 | QKU32221 | QKS66288 | QKE49372 | QJQ82944 | QJD23881 | QIV15064 |
| QKV39751 | QKU32257 | QKS66300 | QKE49384 | QJQ83892 | QJD23893 | QIV15076 |
| QKV39763 | QKU32281 | QKS66312 | QKE49396 | QJQ83952 | QJD23905 | QIV15088 |
| QKV39775 | QKU32293 | QKS66336 | QKE49408 | QJQ83964 | QJD23929 | QIV15100 |
| QKV39787 | QKU32329 | QKS66348 | QKE49420 | QJQ83976 | QJD23941 | QIV15112 |
| QKV39799 | QKU32341 | QKS66360 | QKE49432 | QJQ84060 | QJD23965 | QIV15124 |
| QKV39811 | QKU32353 | QKS66372 | QKE49444 | QJQ84084 | QJD23977 | QIV15136 |
| QKV39823 | QKU32365 | QKS66384 | QKE49456 | QJQ84108 | QJD23989 | QIV15148 |
| QKV39835 | QKU32377 | QKS66396 | QKE49468 | QJQ84144 | QJD24001 | QIV15160 |
| QKV39847 | QKU32389 | QKS66408 | QKE49480 | QJQ84156 | QJD24013 | QIV15172 |
| QKV39859 | QKU32401 | QKS66420 | QKE49492 | QJQ84168 | QJD24037 | QIV15184 |
| QKV39871 | QKU32413 | QKS66432 | QKE49504 | QJQ84180 | QJD24049 | QIV15196 |
| QKV39883 | QKU32425 | QKS66444 | QKE49516 | QJQ84192 | QJD24061 | QIU80933 |
| QKV39895 | QKU32437 | QKS66456 | QKE49528 | QJQ84204 | QJD24073 | QIU80957 |
| QKV39907 | QKU32449 | QKS66468 | QKE49552 | QJQ84216 | QJD24085 | QIU80969 |
| QKV39919 | QKU32461 | QKS66480 | QKE49660 | QJQ84228 | QJD24097 | QIU80981 |
| QKV39931 | QKU32473 | QKS66492 | QKE49717 | QJQ84240 | QJD24121 | QIU80993 |
| QKV39943 | QKU32485 | QKS66504 | QKE49729 | QJQ84264 | QJD24133 | QIU81005 |
| QKV39955 | QKU32497 | QKS66516 | QKE49741 | QJQ84276 | QJD24145 | QIU81017 |
| QKV39967 | QKU32509 | QKS66528 | QKE49753 | QJQ84288 | QJD24157 | QIU81029 |
| QKV39979 | QKU32521 | QKS66540 | QKE49765 | QJQ84312 | QJD24169 | QIU81041 |
| QKV39991 | QKU32533 | QKS66552 | QKE49777 | QJQ84324 | QJD24181 | QIU81053 |
| QKV40003 | QKU32545 | QKS66564 | QKE49789 | QJQ84336 | QJD24193 | QIU81065 |
| QKV40015 | QKU32557 | QKS66576 | QKE49801 | QJQ84348 | QJD24205 | QIU81077 |
| QKV40027 | QKU32569 | QKS66588 | QKE49813 | QJQ38368 | QJD24217 | QIU81089 |
| QKV40039 | QKU32581 | QKS66600 | QKE49825 | QJQ38380 | QJD24241 | QIU81101 |
| QKV40051 | QKU32593 | QKS66612 | QKE49837 | QJQ38392 | QJD24253 | QIU81113 |
| QKV40063 | QKU32605 | QKS66624 | QKE49849 | QJQ38404 | QJD24265 | QIU81125 |
| QKV40075 | QKU32617 | QKS66636 | QKE49873 | QJQ38416 | QJD24277 | QIU81137 |
| QKV40087 | QKU32629 | QKS66648 | QKE49885 | QJQ38428 | QJD24289 | QIU81149 |
| QKV40099 | QKU32641 | QKS66660 | QKE49897 | QJQ38440 | QJD24301 | QIU81161 |
| QKV40111 | QKU32653 | QKS66672 | QKE49909 | QJQ38452 | QJD24313 | QIU81173 |
| QKV40123 | QKU32665 | QKS66684 | QKE49921 | QJQ38476 | QJD24337 | QIU81185 |
| QKV40135 | QKU32677 | QKS66696 | QKE49945 | QJQ38488 | QJD24349 | QIU81197 |
| QKV40147 | QKU32689 | QKS66708 | QKE49981 | QJQ38500 | QJD24409 | QIU81209 |
| QKV40159 | QKU32701 | QKS66720 | QKE49993 | QJQ38512 | QJD24433 | QIU81221 |
| QKV40171 | QKU32713 | QKS66732 | QKE50866 | QJQ38524 | QJD24469 | QIU81233 |
| QKV40183 | QKU32725 | QKS66744 | QKE50878 | QJQ38536 | QJD24481 | QIU81245 |
| QKV40195 | QKU32737 | QKS66756 | QKE50890 | QJQ38560 | QJD24493 | QIU81257 |
| QKV40207 | QKU32749 | QKS66768 | QKE50902 | QJQ38572 | QJD24505 | QIU81269 |
| QKV40219 | QKU32773 | QKS66780 | QKE50914 | QJQ38584 | QJD24517 | QIU81281 |
| QKV40231 | QKU32785 | QKS66792 | QKE50926 | QJQ38596 | QJD24529 | QIU81293 |
| QKV40243 | QKU32809 | QKS66804 | QKE50938 | QJQ38608 | QJD24553 | QIU81305 |
| QKV40255 | QKU32821 | QKS66816 | QKE50950 | QJQ38620 | QJD24577 | QIU81317 |
| QKV40267 | QKU32833 | QKS66828 | QKE50962 | QJQ38632 | QJD24589 | QIU81329 |
| QKV40279 | QKU32845 | QKS66840 | QKE50974 | QJQ38644 | QJD24613 | QIU81341 |
| QKV40291 | QKU32857 | QKS66864 | QKE50986 | QJQ38656 | QJD24637 | QIU81353 |
| QKV40303 | QKU32881 | QKS66960 | QKE50998 | QJQ38668 | QJD24661 | QIU81365 |
| QKV40315 | QKU32905 | QKS66972 | QKE51010 | QJQ38680 | QJD24673 | QIU81389 |
| QKV40327 | QKU32941 | QKS66984 | QKE51022 | QJQ38704 | QJD24685 | QIU81401 |
| QKV40339 | QKU32953 | QKS66996 | QKE51034 | QJQ38716 | QJD24697 | QIU81413 |
| QKV40351 | QKU32965 | QKS67008 | QKE53883 | QJQ38728 | QJD24709 | QIU81425 |
| QKV40363 | QKU32977 | QKS67020 | QKE53941 | QJQ38740 | QJD24733 | QIU81437 |
| QKV40375 | QKU32989 | QJD07600 | QKE53953 | QJQ38752 | QJD24745 | QIU81449 |
| QKV40387 | QKU33001 | QJD07612 | QKE53977 | QJQ38764 | QJD24757 | QIU81461 |
| QKV40399 | QKU33013 | QJD07624 | QKE53989 | QJQ38776 | QJD24781 | QIU81473 |
| QKV40411 | QKU33025 | QKQ11697 | QKE54046 | QJQ38788 | QJD24793 | QIU81485 |
| QKV40423 | QKU33037 | QKQ11709 | QKE54058 | QJQ38800 | QJD24805 | QIU81509 |
| QKV40447 | QKU33049 | QKQ11721 | QKE54070 | QJQ38812 | QJD24829 | QIU81521 |
| QKV40459 | QKU33061 | QKQ63408 | QKE54094 | QJQ38824 | QJD24841 | QIU81533 |
| QKV40471 | QKU33073 | QKQ63420 | QKE54106 | QJQ38836 | QJD24853 | QIU81545 |
| QKV40483 | QKU33085 | QKQ63432 | QKE54118 | QJQ38848 | QJD24877 | QIU81557 |
| QKV40495 | QKU33097 | QKQ63444 | QKE54130 | QJQ38860 | QJD24889 | QIU81569 |
| QKV40507 | QKU33109 | QKQ63456 | QKE44649 | QJQ38872 | QJD24901 | QIU81581 |
| QKV40519 | QKU33121 | QKQ63468 | QKE44661 | QJQ38920 | QJD24913 | QIU81593 |
| QKV40531 | QKU33133 | QKQ63480 | QKE44673 | QJQ38932 | QJD24949 | QIU81605 |
| QKV40543 | QKU33145 | QKQ63492 | QKE44685 | QJQ38968 | QJD24961 | QIU81617 |
| QKV40555 | QKU33157 | QKQ63504 | QKE44697 | QJQ38992 | QJD24973 | QIU81641 |
| QKV40567 | QKU33169 | QKQ63516 | QKE44709 | QJQ39028 | QJD25009 | QIU81653 |
| QKV40579 | QKU33181 | QKQ63528 | QKE44721 | QJQ39052 | QJD25021 | QIU81677 |
| QKV40591 | QKU33193 | QKQ63540 | QKE44733 | QJQ39064 | QJD25033 | QIU81689 |
| QKV40603 | QKU33205 | QKQ63552 | QKE44745 | QJQ39088 | QJD25057 | QIU81701 |
| QKV40615 | QKU33217 | QKQ63564 | QKE44757 | QJQ39112 | QJD25069 | QIU81725 |
| QKV40627 | QKU33229 | QKQ63576 | QKE44769 | QJQ39124 | QJD25081 | QIU81749 |
| QKV40639 | QKU33241 | QKQ63588 | QKE44781 | QJQ39136 | QJD25117 | QIU81761 |
| QKV40651 | QKU33253 | QKQ63600 | QKE44793 | QJQ39148 | QJD25129 | QIU81906 |
| QKV40663 | QKU33265 | QKQ63612 | QKE44805 | QJQ39160 | QJD25141 | QIU81918 |
| QKV40675 | QKU33277 | QKQ63624 | QKE44817 | QJQ39172 | QJD25153 | QIT06887 |
| QKV40687 | QKU33289 | QKQ63636 | QKE44829 | QJQ39196 | QJD25165 | QIT06899 |
| QKV40699 | QKU33301 | QKQ63648 | QKE44841 | QJQ39220 | QJD25201 | QIT06911 |
| QKV40711 | QKU33313 | QKQ63660 | QKE44853 | QJQ39244 | QJD25225 | QIT06923 |
| QKV40723 | QKU33325 | QKQ63672 | QKE44865 | QJQ39256 | QJD25237 | QIT06935 |
| QKV40735 | QKU33337 | QKQ63684 | QKE44877 | QJQ39268 | QJD25261 | QIT06947 |
| QKV40747 | QKU33349 | QKQ63696 | QKE44889 | QJQ39280 | QJD25273 | QIT06959 |
| QKV40759 | QKU33361 | QKQ63708 | QKE44901 | QJQ39304 | QJD25285 | QIT06971 |
| QKV40795 | QKU33397 | QKQ63720 | QKE44913 | QJQ39328 | QJD25309 | QIS60306 |
| QKV40807 | QKU33409 | QKQ63732 | QKE44925 | QJQ39340 | QJD25321 | QIS60316 |
| QKV40819 | QKU37173 | QKQ63744 | QKE44937 | QJQ39364 | QJD25333 | QIS60326 |
| QKV40831 | QKU37185 | QKQ63756 | QKE44949 | QJQ39376 | QJD25381 | QIS60336 |
| QKV40843 | QKU37197 | QKQ63768 | QKE44961 | QJQ39400 | QJD25393 | QIS60346 |
| QKV40855 | QKU37209 | QKQ63780 | QKE44973 | QJQ39472 | QJD25405 | QIS60356 |
| QKV40867 | QKU37221 | QKQ63792 | QKE44985 | QJQ39484 | QJD25417 | QIS60366 |
| QKV40879 | QKU37233 | QKQ63804 | QKE44997 | QJQ39508 | QJD25453 | QIS60376 |
| QKV40891 | QKU37245 | QKQ63816 | QKE45009 | QJQ39520 | QJD25465 | QIS60386 |
| QKV40903 | QKU37257 | QKQ63828 | QKE45021 | QJQ39532 | QJD25489 | QIS60396 |
| QKV40915 | QKU37269 | QKQ63840 | QKE45033 | QJQ39568 | QJD25501 | QIS60406 |
| QKV40927 | QKU37281 | QKQ63852 | QKE45045 | QJQ39580 | QJD25513 | QIS60416 |
| QKV40939 | QKU37293 | QKQ63864 | QKE45482 | QJQ39592 | QJD25525 | QIS60426 |
| QKV40951 | QKU37305 | QKQ63876 | QKE45494 | QJQ39604 | QJD25537 | QIS60436 |
| QKV40963 | QKU37329 | QKQ63888 | QKE45506 | QJQ39616 | QJD25573 | QIS60446 |
| QKV40975 | QKU37353 | QKQ63900 | QKE45518 | QJQ39628 | QJD25585 | QIS60456 |
| QKV40987 | QKU37365 | QKQ63912 | QKE45530 | QJQ39640 | QJD25621 | QIS60466 |
| QKV40999 | QKU37389 | QKK14410 | QKE45556 | QJQ39664 | QJD25633 | QIS60476 |
| QKV41011 | QKU37401 | QKO24075 | QKE45568 | QJQ39676 | QJD25645 | QIS60486 |
| QKV41023 | QKU37425 | QKO24087 | QKE45580 | QJQ39688 | QJD25657 | QIS60496 |
| QKV41035 | QKU37449 | QKO24099 | QKE45592 | QJQ39724 | QJD25693 | QIS60506 |
| QKV41047 | QKU37461 | QKO24111 | QKE45604 | QJQ39748 | QJD25705 | QIS60518 |
| QKV41059 | QKU37473 | QKO24123 | QKE45616 | QJQ39760 | QJD25717 | QIS60530 |
| QKV41071 | QKU37521 | QKO24135 | QKE45628 | QJQ39808 | QJD25729 | QIS60542 |
| QKV41083 | QKU37545 | QKO24147 | QKE45640 | QJQ39844 | QJD25741 | QIS60554 |
| QKV41095 | QKU37557 | QKO24159 | QKE45652 | QJQ39856 | QJD25765 | QIS60578 |
| QKV41107 | QKU37569 | QKO24171 | QKE45664 | QJQ39868 | QJC19511 | QIS60590 |
| QKV41119 | QKU37581 | QKO24183 | QKE45688 | QJQ39892 | QJC19523 | QIS60602 |
| QKV41131 | QKU37593 | QKO24206 | QKE45712 | QJQ39904 | QJC19535 | QIS60626 |
| QKV41143 | QKU37605 | QKO24241 | QKE45736 | QJQ39916 | QJC19547 | QIS60638 |
| QKV41155 | QKU37617 | QKO24253 | QKE45772 | QJQ39928 | QJC19559 | QIS60650 |
| QKV41167 | QKU37629 | QKO24265 | QKE45796 | QJQ39940 | QJC19571 | QIS60674 |
| QKV41179 | QKU37641 | QKO24277 | QKE45808 | QJQ27909 | QJC19583 | QIS60686 |
| QKV41191 | QKU37653 | QKO00482 | QKE45820 | QJQ27921 | QJC19595 | QIS60698 |
| QKV41203 | QKU37665 | QKN19535 | QKE45844 | QJQ27933 | QJC19607 | QIS60722 |
| QKV41215 | QKU37689 | QKN19679 | QKE45856 | QJQ27945 | QJC19619 | QIS60734 |
| QKV41239 | QKU37701 | QKN19691 | QKE45868 | QJQ27957 | QJC19631 | QIS60746 |
| QKV41251 | QKU37713 | QKN19751 | QKE45892 | QJQ27969 | QJC19643 | QIS60770 |
| QKV41263 | QKU37725 | QKN19835 | QKE45904 | QJQ27981 | QJC19655 | QIS60782 |
| QKV41275 | QKU37737 | QKN19847 | QKE45916 | QJQ27993 | QJC19667 | QIS60794 |
| QKV41287 | QKU37761 | QKN19883 | QKE45928 | QJQ28005 | QJC19679 | QIS60806 |
| QKV41299 | QKU37809 | QKN19943 | QKE45940 | QJQ28017 | QJC19691 | QIS60818 |
| QKV41311 | QKU37821 | QKN20039 | QKE45952 | QJQ28029 | QJC19703 | QIS60842 |
| QKV41323 | QKU37833 | QKN20099 | QKE45976 | QJQ28041 | QJC19715 | QIS60866 |
| QKV41335 | QKU37845 | QKN20147 | QKE45988 | QJQ28053 | QJC19727 | QIS60878 |
| QKV41347 | QKU37857 | QKN20183 | QKE46000 | QJQ28065 | QJC19739 | QIS60890 |
| QKV41359 | QKU37869 | QKN20219 | QKE46012 | QJQ28077 | QJC19751 | QIS60902 |
| QKV41371 | QKU52841 | QKN20243 | QKE46024 | QJQ28089 | QJC19763 | QIS60914 |
| QKV41383 | QKU52865 | QKN20255 | QKE46036 | QJQ28101 | QJC19775 | QIS60926 |
| QKV41395 | QKU52877 | QKN20291 | QKE46048 | QJQ28113 | QJC19787 | QIS60938 |
| QKV41407 | QKU52889 | QKN20315 | QKE11670 | QJQ28125 | QJC19799 | QIS60950 |
| QKV41419 | QKU52901 | QKN20339 | QKE12102 | QJQ28137 | QJC19811 | QIS60962 |
| QKV41431 | QKU52913 | QKN20411 | QKE22895 | QJQ28149 | QJC19823 | QIS60974 |
| QKV41443 | QKU52925 | QKN20423 | QKE22907 | QJQ28161 | QJC19835 | QIS60986 |
| QKV41455 | QKU52937 | QKN20435 | QKE22919 | QJQ28173 | QJC19847 | QIS60998 |
| QKV41467 | QKU52949 | QKN20447 | QKE22931 | QJQ28185 | QJC19859 | QIS61010 |
| QKV41479 | QKU52961 | QKN20483 | QJY78196 | QJQ28197 | QJC19871 | QIS61022 |
| QKV41491 | QKU52973 | QKN20507 | QJY78208 | QJQ28209 | QJC19883 | QIS61034 |
| QKV41503 | QKU52985 | QKN20519 | QJY78232 | QJQ28221 | QJC19907 | QIS61046 |
| QKV41515 | QKU52997 | QKN20567 | QJY78244 | QJQ28233 | QJC19919 | QIS61058 |
| QKV41527 | QKU53009 | QKN20639 | QJY78279 | QJQ28245 | QJC19931 | QIS61082 |
| QKV41539 | QKU53021 | QKN20687 | QJY78291 | QJQ28257 | QJC19943 | QIS61094 |
| QKV41551 | QKU53033 | QKN20699 | QKC05040 | QJQ28269 | QJC19955 | QIS61106 |
| QKV41563 | QKU53045 | QKN20711 | QKC05052 | QJQ28281 | QJC19967 | QIS61118 |
| QKV41575 | QKU53057 | QKN20723 | QKC05064 | QJQ28293 | QJC19979 | QIS61130 |
| QKV41587 | QKU53069 | QKN20735 | QKC05076 | QJQ28317 | QJC19991 | QIS61142 |
| QKV41599 | QKU53081 | QKN20747 | QKC05088 | QJQ28329 | QJC20003 | QIS61154 |
| QKV41611 | QKU53093 | QKN20759 | QKC05100 | QJQ04264 | QJC20027 | QIS61166 |
| QKV41623 | QKU53105 | QKN20771 | QKC05112 | QJQ04276 | QJC20039 | QIS61178 |
| QKV41635 | QKU53117 | QKN20783 | QKC05124 | QJQ04288 | QJC20051 | QIS61190 |
| QKV41647 | QKU53129 | QKN20795 | QKC05136 | QJQ04300 | QJC20063 | QIS61202 |
| QKV41659 | QKU53141 | QKN20807 | QKC05148 | QJQ04312 | QJC20075 | QIS61214 |
| QKV41671 | QKU53153 | QKN20819 | QKC05160 | QJQ04324 | QJC20087 | QIS61226 |
| QKV41683 | QKU53165 | QKN20831 | QKC05172 | QJQ04336 | QJC20099 | QIS61238 |
| QKV41695 | QKU53177 | QKN20843 | QKC05184 | QJQ04348 | QJC20111 | QIS61250 |
| QKV41707 | QKU53189 | QKN20855 | QKC05196 | QJQ04360 | QJC20123 | QIS61262 |
| QKV41719 | QKU53201 | QKN20867 | QKC05208 | QJQ27005 | QJC20135 | QIS61274 |
| QKV41731 | QKU53213 | QKN20879 | QKC05220 | QJP03559 | QJC20147 | QIS61286 |
| QKV41743 | QKU53225 | QKN20891 | QKC05232 | QJP03571 | QJC20159 | QIS61298 |
| QKV41755 | QKU53237 | QKN20903 | QKC05244 | QJI07194 | QJC20171 | QIS61310 |
| QKV41767 | QKU53249 | QKN20915 | QKC05256 | QJI07206 | QJC20183 | QIS61322 |
| QKV41779 | QKU53261 | QKN20927 | QKC05268 | QJI07218 | QJC20195 | QIS61334 |
| QKV41791 | QKU53273 | QKN20939 | QKC05280 | QJI07230 | QJC20207 | QIS61346 |
| QKV41803 | QKU53285 | QKN20951 | QKC05292 | QJI07242 | QJC20219 | QIS61358 |
| QKV41815 | QKU53297 | QKK14619 | QKC05304 | QJI07254 | QJC20243 | QIS61370 |
| QKV41827 | QKU53321 | QKG86662 | QKC05316 | QJI07266 | QJC20255 | QIS61382 |
| QKV41839 | QKU53333 | QKG86674 | QKC05328 | QJI07278 | QJC20267 | QIS61394 |
| QKV41851 | QKU53345 | QKG86686 | QKC05340 | QJI07290 | QJC20279 | QIS61406 |
| QKV41863 | QKU53357 | QKG86710 | QKC05352 | QJI07302 | QJC20291 | QIS61418 |
| QKV41875 | QKU53369 | QKG86722 | QKC05364 | QJI07314 | QJC20303 | QIS61430 |
| QKV41887 | QKU53381 | QKG86734 | QKC05376 | QJI07326 | QJC20315 | QIS61442 |
| QKV41899 | QKU53393 | QKG86758 | QKC05388 | QJI07338 | QJC20327 | QIS61454 |
| QKV41911 | QKU53405 | QKG86770 | QKC05400 | QJI54046 | QJC20339 | QIS61490 |
| QKV41923 | QKU53417 | QKG86782 | QKC05412 | QJI54058 | QJC20351 | QIS61502 |
| QKV41935 | QKU53429 | QKG86794 | QKC60843 | QJI54070 | QJC20363 | QIS61514 |
| QKV41947 | QKU53441 | QKG86806 | QKC60855 | QJI54082 | QJC20375 | QIS61526 |
| QKV41971 | QKU53453 | QKG86818 | QJY51512 | QJI54106 | QJC20387 | QIS61550 |
| QKV41983 | QKU53465 | QKG86830 | QJY51572 | QJI54118 | QJC20399 | QIS61562 |
| QKV41995 | QKU53477 | QKG86842 | QJY51680 | QJI54130 | QJC20411 | QIS61574 |
| QKV42007 | QKU53489 | QKG86854 | QJY51704 | QJI54142 | QJC20423 | QIS30072 |
| QKV42019 | QKU53501 | QKG86866 | QJY51908 | QJI54166 | QJC20435 | QIS30082 |
| QKV42031 | QKU53513 | QKG86890 | QJY51920 | QJI54189 | QJC20447 | QIS30092 |
| QKV42043 | QKU53525 | QKG86902 | QJY39889 | QJI54201 | QJC20459 | QIS30102 |
| QKV42055 | QKU53537 | QKG86914 | QJY39901 | QJI54249 | QJC20471 | QIS30112 |
| QKV42067 | QKU53549 | QKG86926 | QJY39913 | QJI54261 | QJC20483 | QIS30122 |
| QKV42079 | QKU53561 | QKG86938 | QJY39925 | QJI54307 | QJC20495 | QIS30132 |
| QKV42091 | QKU53573 | QKG86950 | QJY39937 | QJI54331 | QJC20506 | QIS30162 |
| QKV42103 | QKU53585 | QKG86962 | QJY39949 | QJI54343 | QJC20518 | QIS30172 |
| QKV42115 | QKU53597 | QKG86974 | QJY39961 | QJI54415 | QJC20530 | QIS30182 |
| QKV42127 | QKU53609 | QKG86986 | QJY39973 | QJF75139 | QJC20542 | QIS30202 |
| QKV42139 | QKU53621 | QKG86998 | QJY39985 | QJF75319 | QJC20554 | QIS30212 |
| QKV42151 | QKU53633 | QKG87010 | QJY39997 | QJF75355 | QJC20566 | QIS30222 |
| QKV42163 | QKU53645 | QKG87034 | QJY40009 | QJF75391 | QJC20578 | QIS30242 |
| QKV42175 | QKU53657 | QKG87046 | QJY40021 | QJF75403 | QJC20590 | QIS30252 |
| QKV42199 | QKU53669 | QKG87058 | QJY40033 | QJF75415 | QJC20602 | QIS30262 |
| QKV42211 | QKU53681 | QKG87070 | QJY40045 | QJF75991 | QJC20614 | QIS30272 |
| QKV42223 | QKU53693 | QKG87082 | QJY40057 | QJF76003 | QJC20626 | QIS30282 |
| QKV42235 | QKU53705 | QKG87094 | QJY40069 | QJF76015 | QJC20638 | QIS30292 |
| QKV42247 | QKU53717 | QKG87106 | QJY40081 | QJF76027 | QJC20650 | QIS30302 |
| QKV42259 | QKU53729 | QKG87118 | QJY40117 | QJF76039 | QJC20662 | QIS30312 |
| QKV42271 | QKU53741 | QKG87142 | QJY40129 | QJF76075 | QJC20674 | QIS30322 |
| QKV42283 | QKU53753 | QKG87154 | QJY40153 | QJF76087 | QJC20686 | QIS30332 |
| QKV42295 | QKU53777 | QKG87166 | QJY40165 | QJF76099 | QJC20698 | QIS30342 |
| QKV42307 | QKU53789 | QKG87178 | QJY40189 | QJF76111 | QJC20710 | QIS30352 |
| QKV42319 | QKU53801 | QKG87190 | QJY40225 | QJF76123 | QJC20722 | QIS30372 |
| QKV42331 | QKU53813 | QKG87202 | QJY40297 | QJF76135 | QJC20734 | QIS30382 |
| QKV42343 | QKU53825 | QKG87214 | QJY40321 | QJF76147 | QJC20746 | QIS30392 |
| QKV42366 | QKU53837 | QKG87226 | QJY40333 | QJF76159 | QJC20758 | QIS30412 |
| QKV42378 | QKU53849 | QKG87238 | QJY40345 | QJF76171 | QJC21037 | QIS30422 |
| QKV42390 | QKU53861 | QKG87250 | QJY40357 | QJF76183 | QJC21049 | QIS30432 |
| QKV42402 | QKU53873 | QKG87262 | QJY40369 | QJF76195 | QJA41661 | QIS30442 |
| QKV42414 | QKU53885 | QKG87274 | QJY40381 | QJF76207 | QJA41673 | QIS30452 |
| QKV42426 | QKU53897 | QKG87286 | QJX68390 | QJF76219 | QJA41685 | QIS30472 |
| QKV42438 | QKU53909 | QKG87298 | QJX68414 | QJF76231 | QJA41697 | QIS30482 |
| QKV42462 | QKU53921 | QKG87334 | QJX68438 | QJF76243 | QJA41709 | QIS30492 |
| QKV42474 | QKU53933 | QKG87346 | QJX68486 | QJF76255 | QJA41721 | QIS30502 |
| QKV42486 | QKU53945 | QKG87358 | QJX68630 | QJF76267 | QJA41733 | QIS30522 |
| QKV42498 | QKU53957 | QKG87370 | QJX68806 | QJF76279 | QJA41745 | QIS30542 |
| QKV42510 | QKU53969 | QKG87382 | QJX68818 | QJF76291 | QJA41757 | QIS30552 |
| QKV42522 | QKU53981 | QKG87394 | QJX68830 | QJF76303 | QJA41769 | QIS30562 |
| QKV42534 | QKU54005 | QKG87406 | QJX68842 | QJF76315 | QJA41781 | QIS30582 |
| QKV42546 | QKU54017 | QKG87418 | QJX68854 | QJF76327 | QJA41793 | QIS30592 |
| QKV42558 | QKU54029 | QKG87430 | QJX68866 | QJF76339 | QJA41805 | QIS30612 |
| QKV42582 | QKU54041 | QKG87442 | QJX68890 | QJF76351 | QJA41817 | QIS30622 |
| QKV42594 | QKU54065 | QKG87454 | QJX68914 | QJF76363 | QJA41829 | QIS30632 |
| QKV42606 | QKU54077 | QKG87466 | QJX68926 | QJF76375 | QJA41841 | QIS30652 |
| QKV42618 | QKU54089 | QKG87478 | QJX68938 | QJF76387 | QJA41853 | QIS30662 |
| QKV42630 | QKU54101 | QKG87514 | QJX68974 | QJF76410 | QJA41865 | QIS30672 |
| QKV42642 | QKU54113 | QKG87526 | QJX68998 | QJF76422 | QJA41993 | QIS30682 |
| QKV42654 | QKU54125 | QKG87538 | QJX69226 | QJF76434 | QJA42005 | QIQ68471 |
| QKV42666 | QKU54137 | QKG87550 | QJX69238 | QJF76446 | QJA42017 | QIQ68481 |
| QKV42678 | QKU54149 | QKG87562 | QJX69250 | QJF76482 | QJA42029 | QIQ68491 |
| QKV42690 | QKU54161 | QKG87574 | QJX69262 | QJF76494 | QJA42041 | QIQ68501 |
| QKV42702 | QKU54173 | QKG87586 | QJX69286 | QJF76506 | QJA42053 | QIQ68511 |
| QKV42714 | QKU54185 | QKG87598 | QJX69298 | QJF76530 | QJA42065 | QIQ68521 |
| QKV42726 | QKU54197 | QKG87610 | QJX69310 | QJF76542 | QJA42077 | QIQ68531 |
| QKV42738 | QKU54209 | QKG87622 | QJX69322 | QJF76554 | QJA42089 | QIQ68541 |
| QKV42750 | QKU54221 | QKG87634 | QJX69418 | QJF76566 | QJA42101 | QIQ68551 |
| QKV42762 | QKU54233 | QKG87646 | QJX69575 | QJF76578 | QJA42113 | QIQ49769 |
| QKV42774 | QKU54245 | QKG87658 | QJX69587 | QJF76590 | QJA42125 | QIQ49779 |
| QKV42786 | QKU54257 | QKG87670 | QJX69647 | QJF76602 | QJA42137 | QIQ49789 |
| QKV42798 | QKU54269 | QKG87682 | QJX69659 | QJF76614 | QJA42149 | QIQ49799 |
| QKV42810 | QKU54281 | QKG87694 | QJX69671 | QJF76626 | QJA42161 | QIQ49809 |
| QKV42822 | QKU54293 | QKG87706 | QJX69683 | QJF76638 | QJA42173 | QIQ49819 |
| QKV42834 | QKU54305 | QKG87718 | QJX69695 | QJF76650 | QJA42185 | QIQ49829 |
| QKV42846 | QKU54317 | QKG87730 | QJX69719 | QJF76674 | QJA42197 | QIQ49839 |
| QKV42858 | QKU54329 | QKG87742 | QJX69767 | QJF76686 | QJA42209 | QIQ49849 |
| QKV42870 | QKU54341 | QKG87754 | QJX69779 | QJF76698 | QJA42221 | QIQ49859 |
| QKV42882 | QKU54365 | QKG87766 | QJX69791 | QJF76710 | QJA42233 | QIQ49869 |
| QKV42894 | QKU54377 | QKG87778 | QJX69827 | QJF76722 | QJA42245 | QIQ49879 |
| QKV42906 | QKU54389 | QKG87790 | QJX69839 | QJF76734 | QJA16408 | QIQ49889 |
| QKV42918 | QKU54401 | QKG87826 | QJX69863 | QJF76746 | QJA16420 | QIQ49899 |
| QKV42930 | QKU54413 | QKG87838 | QJX69887 | QJF76758 | QJA16444 | QIQ49909 |
| QKV42942 | QKU54425 | QKG87850 | QJX69911 | QJF76770 | QJA16456 | QIQ49919 |
| QKV42954 | QKU54437 | QKG87862 | QJX69959 | QJF76782 | QJA16468 | QIQ49939 |
| QKV42966 | QKU54449 | QKG87874 | QJX69971 | QJF76794 | QJA16492 | QIQ49949 |
| QKV42978 | QKU54461 | QKG87910 | QJX69995 | QJF76806 | QJA16504 | QIQ49959 |
| QKV42990 | QKU54473 | QKG87922 | QJX70019 | QJF76818 | QJA16528 | QIQ49969 |
| QKV43002 | QKU54497 | QKG87934 | QJX70031 | QJF76830 | QJA16540 | QIQ49979 |
| QKV43014 | QKU54509 | QKG87946 | QJX70043 | QJF76842 | QJA16564 | QIQ49989 |
| QKV43026 | QKU54521 | QKG87958 | QJX70067 | QJF76854 | QJA16576 | QIQ49999 |
| QKV43038 | QKU54533 | QKG87970 | QJX70091 | QJF76866 | QJA16600 | QIQ50009 |
| QKV43050 | QKV05895 | QKG87994 | QJX70115 | QJF76878 | QJA16612 | QIQ50019 |
| QKV43062 | QKV05931 | QKG88006 | QJX70139 | QJF76890 | QJA16624 | QIQ50029 |
| QKV43074 | QKV05943 | QKG88018 | QJX70151 | QJF76902 | QJA16636 | QIQ50039 |
| QKV43086 | QKV05955 | QKG88042 | QJX70187 | QJF76914 | QJA16660 | QIQ50049 |
| QKV43098 | QKV05967 | QKG88054 | QJX70199 | QJF76926 | QJA16684 | QIQ50059 |
| QKV43110 | QKV05979 | QKG88066 | QJX70211 | QJF76938 | QJA16696 | QIQ50069 |
| QKV43122 | QKV06003 | QKG88078 | QJX70223 | QJF76950 | QJA16708 | QIQ50079 |
| QKV43134 | QKV06015 | QKG88090 | QJX70235 | QJF76962 | QJA16720 | QIQ50089 |
| QKV43146 | QKV06027 | QKG88102 | QJX70271 | QJF76974 | QJA16732 | QIQ50099 |
| QKV43158 | QKV06039 | QKG88138 | QJX70283 | QJF76986 | QJA16744 | QIQ50119 |
| QKV25725 | QKV06051 | QKG88150 | QJX70308 | QJF76998 | QJA16756 | QIQ50129 |
| QKV26522 | QKV06063 | QKG88174 | QJX70335 | QJF77010 | QJA16768 | QIQ50139 |
| QKV26546 | QKV06075 | QKG88186 | QJX70347 | QJF77022 | QJA16780 | QIQ50149 |
| QKV26570 | QKV06087 | QKG88198 | QJX70359 | QJF77034 | QJA16792 | QIQ50159 |
| QKV26582 | QKV06099 | QKG88210 | QJX70371 | QJF77046 | QJA16816 | QIQ50169 |
| QKV26594 | QKV06111 | QKG88222 | QJX70383 | QJF77070 | QJA16828 | QIQ50179 |
| QKV26606 | QKV06123 | QKG88246 | QJX70395 | QJF77082 | QJA16840 | QIQ50189 |
| QKV26618 | QKV06135 | QKG88258 | QJX70407 | QJF77094 | QJA16852 | QIQ50199 |
| QKV26630 | QKV06147 | QKG88270 | QJX70419 | QJF77106 | QJA16864 | QIK50435 |
| QKV26642 | QKV06159 | QKG88282 | QJX70431 | QJF77130 | QJA16888 | QIK02951 |
| QKV26654 | QKV06171 | QKG88294 | QJX70443 | QJF77142 | QJA16912 | QIK02961 |
| QKV26666 | QKV06183 | QKG88306 | QJX70455 | QJF77154 | QJA16924 | QIK02971 |
| QKV26678 | QKV06195 | QKG88318 | QJX70467 | QJF77166 | QJA16936 | QIJ96470 |
| QKV26690 | QKV06207 | QKG88342 | QJX70479 | QJF77178 | QJA16948 | QIJ96480 |
| QKS89851 | QKV06219 | QKG88354 | QJX70491 | QJF77190 | QJA16960 | QIJ96490 |
| QKS89887 | QKV06231 | QKG88366 | QJX70503 | QJF77202 | QJA16984 | QIJ96500 |
| QKS89899 | QKV06243 | QKG88378 | QJX70515 | QJF77214 | QJA16996 | QIJ96510 |
| QKS89923 | QKV06255 | QKG88390 | QJX70527 | QJF77226 | QJA17008 | QIJ96520 |
| QKS89947 | QKV06267 | QKG88402 | QJX70539 | QJF77238 | QJA17020 | QIJ96530 |
| QKS89971 | QKV06279 | QKG88414 | QJX70551 | QJF77250 | QJA17031 | QII87790 |
| QKS89995 | QKV06291 | QKG88426 | QJX70563 | QJF77262 | QJA17044 | QII87802 |
| QKS90007 | QKV06303 | QKG88438 | QJX70575 | QJF77274 | QJA17056 | QII87814 |
| QKS90019 | QKV06315 | QKG88450 | QJX70587 | QJF77286 | QJA17068 | QII87826 |
| QKS90031 | QKV06327 | QKG88474 | QJX70599 | QJF77310 | QJA17080 | QII87838 |
| QKS90043 | QKV06339 | QKG88486 | QJX70690 | QJF77322 | QJA17092 | QII57175 |
| QKS90067 | QKV06351 | QKG88498 | QJX70702 | QJG65708 | QJA17104 | QII57185 |
| QKS90079 | QKV06363 | QKG88522 | QJX70714 | QJG65720 | QJA17116 | QII57195 |
| QKS90091 | QKV06375 | QKG88534 | QJX70726 | QJG65732 | QJA17128 | QII57205 |
| QKS90103 | QKV06387 | QKG88546 | QJX70738 | QJG65744 | QJA17140 | QII57215 |
| QKS90115 | QKV06399 | QKG88558 | QJX70750 | QJG65756 | QJA17152 | QII57225 |
| QKS90127 | QKV06423 | QKG88570 | QJX74543 | QJG65768 | QJA17164 | QII57235 |
| QKS90139 | QKV06435 | QKG88582 | QJX74555 | QJG65780 | QJA17176 | QII57245 |
| QKS90151 | QKV06447 | QKG88594 | QJX44847 | QJG65792 | QJA17212 | QII57255 |
| QKS90175 | QKV06459 | QKG88630 | QJX44859 | QJG65804 | QJA17224 | QII57265 |
| QKS90187 | QKV06471 | QKG88642 | QJX44871 | QJG65816 | QJA17236 | QII57275 |
| QKS90199 | QKV06483 | QKG88654 | QJX44883 | QJG65828 | QJA17248 | QII57285 |
| QKS90211 | QKV06495 | QKG88666 | QJX44895 | QJG65840 | QJA17260 | QII57295 |
| QKS90223 | QKV06507 | QKG88678 | QJX44907 | QJG65852 | QJA17272 | QII57305 |
| QKS90235 | QKV06519 | QKG88690 | QJX44919 | QJG65864 | QJA17283 | QII57315 |
| QKS90247 | QKV06531 | QKG88702 | QJX44931 | QJG65876 | QJA17296 | QII57325 |
| QKS90259 | QKV06543 | QKG88714 | QJX44943 | QJG65888 | QJA17308 | QII57335 |
| QKS90271 | QKV06555 | QKG88726 | QJX44955 | QJG65900 | QJA17320 | QII57345 |
| QKS90283 | QKV06567 | QKG88738 | QJX44967 | QJG65912 | QJA17332 | QIH55228 |
| QKS90295 | QKV06579 | QKG88750 | QJX44979 | QJG65924 | QJA17344 | QID98801 |
| QKS90307 | QKV06591 | QKG88762 | QJX44991 | QJF11955 | QJA17356 | QID21055 |
| QKS90319 | QKV06603 | QKG88774 | QJX45003 | QJF11967 | QJA17368 | QID21065 |
| QKS90331 | QKV06615 | QKG88786 | QJX45015 | QJF11979 | QJA17380 | QID21075 |
| QKS90343 | QKV06627 | QKG88798 | QJX45027 | QJF11991 | QJA17392 | QHZ87589 |
| QKS90355 | QKV06639 | QKG88810 | QJX45039 | QJE37823 | QJA17404 | QHZ87599 |
| QKS90367 | QKV06651 | QKG88822 | QJX45051 | QJE37835 | QJA17416 | QHZ00396 |
| QKS90379 | QKV06663 | QKG88834 | QJX45063 | QJE37847 | QJA17428 | QHZ00406 |
| QKS90391 | QKV06675 | QKG88858 | QJX45075 | QJE37859 | QJA17439 | QHW06046 |
| QKS90403 | QKV06687 | QKG88870 | QJX45087 | QJE37871 | QJA17452 | QHW06056 |
| QKS90415 | QKV06699 | QKG88882 | QJX45099 | QJE37883 | QJA17464 | QHW06066 |
| QKS90427 | QKV06711 | QKG88894 | QJX45111 | QJE37895 | QJA17476 | QHU79201 |
| QKS90439 | QKV06723 | QKG88906 | QJX45123 | QJE38218 | QJA17488 | QHU79211 |
| QKS90451 | QKV06735 | QKG88918 | QJX45135 | QJE38230 | QJA17532 | QHQ71970 |
| QKS90463 | QKV06747 | QKG88930 | QJX45147 | QJE38242 | QJA17544 | QHQ71980 |
| QKS90475 | QKV06759 | QKG88942 | QJX45159 | QJE38254 | QJA17556 | QHQ82471 |
| QKS90487 | QKV06771 | QKG88954 | QJX45171 | QJE38266 | QJA17568 | QHO62884 |
| QKS90499 | QKV06783 | QKG88966 | QJX45183 | QJE38278 | QJA17580 | QHO60601 |
| QKS90511 | QKV06795 | QKG88978 | QJX45195 | QJE38290 | QJA17592 | |
| QKS90523 | QKV06807 | QKG88990 | QJX45207 | QJE38302 | QJA17604 | |
| QKS90535 | QKV06819 | QKG89002 | QJX45219 | QJE38314 | QJA17616 | |
| QKS90547 | QKV06831 | QKG89014 | QJX45231 | QJE38326 | QJA17628 | |
| QKS90559 | QKV06843 | QKG89026 | QJX45243 | QJE38338 | QJA17640 | |
| QKS90571 | QKV06855 | QKG89038 | QJX45255 | QJE38350 | QJA17652 | |
| QKS90583 | QKV06867 | QKG89050 | QJX45267 | QJE38362 | QJA17664 | |
| QKS90595 | QKV06879 | QKG89062 | QJX45279 | QJE38374 | QJA17676 | |
| QKS90607 | QKV06891 | QKG89074 | QJW39948 | QJE38386 | QJA17688 | |
| QKS90619 | QKV06903 | QKG89086 | QJU70229 | QJE38398 | QJA17700 | |
| QKS90631 | QKV06915 | QKG89098 | QJU70241 | QJE38410 | QJA17712 | |
| QKS90643 | QKV06927 | QKG89110 | QJU70253 | QJE38422 | QJA17724 | |
| QKS90655 | QKV06939 | QKG89122 | QJU70265 | QJE38434 | QJA17736 | |
| QKS90667 | QKV06951 | QKG89146 | QJU70277 | QJE38446 | QJA17748 | |
| QKS90679 | QKV06963 | QKG89158 | QJU70289 | QJE38458 | QJA17760 | |
| QKS90691 | QKV06975 | QKG89170 | QJU70301 | QJE38470 | QIZ97047 |  |
| QKS90703 | QKV06987 | QKG89182 | QJU70313 | QJE38482 | QIZ97058 |  |
| QKS90715 | QKV06999 | QKG89194 | QJU70325 | QJE38494 | QIZ97070 |  |
| QKS90727 | QKV07011 | QKG89206 | QJU70337 | QJE38506 | QIZ64286 |  |
| QKS90739 | QKV07023 | QKG89218 | QJU70349 | QJE38518 | QIZ64322 |  |
| QKS90751 | QKV07035 | QKG89230 | QJU70361 | QJE38530 | QIZ64358 |  |
| QKS90763 | QKV07059 | QKG89242 | QJU70373 | QJE38542 | QIZ64382 |  |
| QKS90775 | QKV07071 | QKG89254 | QJU70385 | QJE38554 | QIZ64406 |  |
| QKS90787 | QKV07083 | QKG89266 | QJU70397 | QJE38566 | QIZ64418 |  |
| QKS90799 | QKV07095 | QKG89278 | QJU70409 | QJE38578 | QIZ64430 |  |
| QKS90811 | QKV07107 | QKG89290 | QJU70421 | QJE38590 | QIZ64442 |  |
| QKS90823 | QKV07119 | QKG89302 | QJU70433 | QJE38602 | QIZ64454 |  |
| QKS90835 | QKV07131 | QKG89326 | QJU70445 | QJE38614 | QIZ64466 |  |
| QKS90847 | QKV07143 | QKG89350 | QJU70457 | QJE38626 | QIZ64478 |  |
| QKS90859 | QKV07155 | QKG89362 | QJU70469 | QJE38638 | QIZ64490 |  |
| QKS90871 | QKV07167 | QKG89374 | QJU70481 | QJE38650 | QIZ64502 |  |
| QKS90883 | QKV07191 | QKG89386 | QJU70493 | QJE38662 | QIZ64514 |  |
| QKS90895 | QKV07203 | QKG89398 | QJU70505 | QJE38674 | QIZ64538 |  |
| QKS90919 | QKV07215 | QKG89410 | QJU70517 | QJE38686 | QIZ64562 |  |
| QKS90931 | QKV07227 | QKG89422 | QJU70529 | QJE38698 | QIZ64574 |  |
| QKS90943 | QKV07239 | QKG89434 | QJU70541 | QJE38710 | QIZ64693 |  |
